# Insulin secretory and antidiabetic actions of *Heritiera fomes* bark together with isolation of active phytomolecules

**DOI:** 10.1371/journal.pone.0264632

**Published:** 2022-03-03

**Authors:** Prawej Ansari, Peter R. Flatt, Patrick Harriott, Yasser H. A. Abdel-Wahab

**Affiliations:** 1 School of Biomedical Sciences, Ulster University, Coleraine, Co. Londonderry, Northern Ireland, United Kingdom; 2 Department of Pharmacy, School of Pharmacy and Public Health, Independent University, Dhaka, Bangladesh; Medical University of Vienna, AUSTRIA

## Abstract

In folklore, *Heritiera fomes* (*H*. *fomes*) has been extensively used in treatment of various ailments such as diabetes, cardiac and hepatic disorders. The present study aimed to elucidate the antidiabetic actions of hot water extract of *H*. *fomes* (HWHF), including effects on insulin release from BRIN BD11 cells and isolated mouse islets as well as glucose homeostasis in high-fat-fed rats. Molecular mechanisms underlying anti-diabetic activity along with isolation of active compounds were also evaluated. Non-toxic concentrations of HWHF stimulated concentration-dependent insulin release from isolated mouse islets and clonal pancreatic β-cells. The stimulatory effect was potentiated by glucose and isobutyl methylxanthine (IBMX), persisted in presence of tolbutamide or a depolarizing concentration of KCl but was attenuated by established inhibitors of insulin release such as diazoxide, verapamil, and Ca^2+^ chelation. HWHF caused depolarization of the β-cell membrane and increased intracellular Ca^2+^. The extract also enhanced glucose uptake and insulin action in 3T3-L1 differentiated adipocytes cells and significantly inhibited in a dose-dependent manner starch digestion, protein glycation, DPP-IV enzyme activity, and glucose diffusion *in vitro*. Oral administration of HWHF (250 mg/5ml/kg b.w.) to high-fat fed rats significantly improved glucose tolerance and plasma insulin responses and it inhibited plasma DPP-IV activity. HWHF also decreased *in vivo* glucose absorption and intestinal disaccharidase activity while increasing gastrointestinal motility and unabsorbed sucrose transit. Compounds were isolated from HWHF with similar molecular weights to quercitrin (C_21_ H_20_ O_11_) ranging from 447.9 to 449.9 Da which stimulated the insulin release *in vitro* and improved both glucose tolerance and plasma insulin responses in mice. In conclusion, *H*. *fomes* and its water-soluble phytochemicals such as quercitrin may exert antidiabetic actions mediated through a variety of mechanisms which might be useful as dietary adjunct in the management of type 2 diabetes.

## Introduction

Metabolic syndrome is characterized by glucose intolerance, hypertension, dyslipidaemia, and central obesity [1]. The aetiology of the metabolic syndrome includes an altered inflammatory state, visceral adipose tissue abnormalities, and sympathetic nervous system activation [2–4]. Other classical markers including elevated triglycerides, HDL-cholesterol, blood pressure, insulin resistance and fasting plasma glucose (FPG), are proposed as possible predictors of diabetes mellitus and cardiovascular disease risk. Diabetes mellitus (DM) is an increasingly common metabolic disorder characterised by complete or relative deficiencies of insulin secretion and action, that culminates in chronic hyperglycaemia [5]. The disease is categorised into two major types including type I which is often associated with complete β-cell destruction. In contrast, type II diabetes is associated with β-cell loss, defective insulin secretion and insulin resistance [6]. Recent statistics on diabetes throughout the world indicate that approximately 90% of patients are suffering from T2DM [7]. It is also well known that chronic hyperglycaemia leads to secondary complications including neuropathy, retinopathy, nephropathy, and cardiovascular disease which constitute a major challenge for health systems [8, 9].

Type 2 diabetes is strongly associated with obesity, advancing age and some hereditary disposition [10]. Diet and physical activity resulting in weight loss are advocated strongly for therapy but often fail to deliver and restore good blood glucose control. Several class of antidiabetic drugs such as metformin, sulphonylureas, meglitinides, thiazolidinediones, GLP-1 mimetics, DPP-IV inhibitors or SGLT2 inhibitors are required to achieve adequate diabetes control [11]. However, these antidiabetic drugs have adverse side effects and are not always affordable to all the sections of society. Thus, there are significant demands in some parts of the world for cheaper, safer, and more accessible alternatives to off-set the burden of diabetes. Many rural areas of developing countries, rely on traditional antidiabetic plant treatments which are used as adjunct therapy for the management of diabetes [12].

*Heritiera fomes* Buch. Ham. (Syn.: *Heritiera minor* or *Amygdalus minor*) is an evergreen moderate size tree growing abundantly in Sundarbans [13]. It is a well-known mangrove species that has been used in alternate medicine especially in Ayurveda since ancient times [13]. Thus, traditional medicine practitioners have used this plant extensively over the years for treating various ailments such as diarrhoea, dysentery, constipation, indigestion, skin diseases such as dermatitis, rash, eczema, boils, itch, scabies, sores, infections as well as hepatic disorders like jaundice, hepatitis [13]. Previous studies on *H*. *fomes* have demonstrated pharmacological and biological benefits in diabetes, cardiovascular disease and goitre [14], and for use as insect repellent [15], wound healer [16], antioxidant [17, 18], antinociceptive, antimicrobial and anticancer agent [14].

Although *H*. *fomes* possess significant therapeutic potential, there is limited available literature regarding its mechanism of action or nature of active compounds that may possibly be responsible for such desirable activities. However, *H*. *fomes* is known to contain many potentially important phytochemicals, including 39.45% polyphenols, 21.12% tannins, 0.25% chlorophyll a, 0.11% carotenoids, 0.09% chlorophyll b, reducing sugars, saponins, alkaloids, glycosides, tannins, steroids, flavonoids, and gums, proanthocyanidins, procyanidins. trimeric, pentameric, hexameric procyanidins, β-Sitosterol, stigmasterol and stigmast-4-en-3-one [19]. The present study was designed to evaluate anti-diabetic activity of *H*. *fomes* and possible active phytochemicals, to elucidate the mechanism of action using different *in vitro* and *in vivo* systems.

## Materials and methods

### Collection and preparation of plant extracts

*H*. *fomes* bark was acquired by Botany Department, Jahangirnagar University, Dhaka, Bangladesh (botanical accession number 43206) from mangrove forest, Khulna, Bangladesh. The bark was processed for hot water extraction as described previously [20]. Twenty-five grams of dry bark powder were added to 1L water and heated until boiling. The decoction was allowed to stand approximately 15 min and solid material was separated using filters (Whatman no. 1). The final extracted semi-solid, sticky residue of *H*. *fomes* bark (denoted HWHF) was freeze-dried using freeze dryer (Varian 801 LY-3-TT, Varian, Lexington, MA, USA) and stored at 4 °C until use. We used a single large scale extraction to generate material for use in all the reported in this paper. Accordingly, there was no variability in efficiency of extraction.

### In vitro insulin-releasing studies

Clonal pancreatic β-cells (BRIN-BD11 cells) represent an insulin-secreting cell line that was produced by electrofusion of primary beta cells from New England Deaconess Hospital rat pancreatic islets with immortal RINm5F cells [21]. The insulin-release studies using BRIN-BD11 cells and isolated mouse islets were performed as per previous descriptions [22]. HWHF was incubated with/without known insulin secretion modulators at different glucose concentrations (1.1, 5.6 or 16.7 mM) for 20 min at 37 °C. Islets were isolated from the pancreatic tissues of mice (40–50 gm, b.w.) by digestion with Collagenase P (Sigma-Aldrich, Dorset, UK). Islets were cultured for 48–72 h in RPMI-1640 and used for insulin release studies as previously reported [22]. Insulin release and insulin content of islet acid ethanol extracts were measured by insulin radioimmunoassay [23]. Detailed methodology is given in (Materials and Methods section: S1.2 and S1.3 in S1 File).

### Membrane potential and intracellular calcium ([Ca^2+^]_i_)

BRIN-BD11 cells were treated with HWHF and changes in membrane potential and intracellular [Ca^2+^]i; were measured using FLIPR membrane potential and calcium assay kits (Molecular Devices, Sunnyvale, CA, USA) according to the manufacturer instructions [20]. BRIN-BD11 cells were seeded in 96 well plates, and left to attach overnight at 37 °C. The media was then removed, and the cells were pre-incubated with KRB 5·6 mM glucose buffer for 10 min at 37°C. Subsequently, cells were treated for 60 min with the FLIPR membrane potential or calcium dye., Changes in signal intensity were measured using a FlexStation 3 microplate reader with an excitation, emission, and cut-off wavelengths of 530 nm, 565 nm, and 550 nm for membrane potential and 485 nm, 525 nm, and 515 nm for intracellular calcium respectively.

### Glycation of insulin

The effects of HWHF on insulin glycation were assessed as described previously using aminoguanidine as positive control [20]. D-glucose (246.5 mM) was incubated with human insulin (1 mg/ml) and NaBH_3_CN (85.3 mg/ml) in absence (control) and presence of HWHF. After 24 h, the reaction was stopped with 0.5 M acetic acid and glycated and non-glycated insulins were quantified by reversed-phase high-performance liquid chromatography (RP-HPLC) [24]. To separate the glycated and non-glycated insulin, 200 μl of reaction mixture were loaded into a (250 × 4.6 mm) Vydac (C-18) analytical column (The Separations Group, California, USA) and eluted at a flow rate of 1 ml/min. The mobile phase was composed of solvent A (0.12% (v/v) TFA/H_2_O) and solvent B (0.1% (v/v) TFA in 70% acetonitrile + 29.9% H_2_O). A linear gradient of 0–35% (v/v) acetonitrile over 10 min, followed by 35–56% (v/v) acetonitrile over 20 min, ending with 56–70% acetonitrile over 5 min. was established to separate the glycated and non-glycated insulin. Elution profiles were observed at 214 and 208 nm. Glycated insulin peak areas were expressed as a percentage of the total glycated and non-glycated peak areas.

### Cellular glucose uptake

Adipocytes differentiated from 3T3L1 fibroblast monolayer cells were incubated with HWHF extract in presence or absence of 100 nM insulin at 37 °C for 30 min in 5% CO_2_ and 95% air atmosphere, before adding 2-NBDG, 2-(N-(7-Nitrobenz-2-oxa-1,3-diazol-4-yl) Amino)-2-Deoxyglucose (50 nM). Cells were washed with ice-cold PBS and three to four coverslips were mounted on slides. Magnified images were taken on the fluorescence microscope to measure glucose uptake as indicated by the fluorescence intensity as described before [20]. Detailed methodology is given in (Materials and Methods section: S1.4 in S1 File).

### DPP-IV enzyme activity *in vitro*

The effects of HWHF on DPP-IV activity were assessed by implementing a fluorometric method using the substrate Gly-Pro-AMC as documented previously [25]. The activity of the enzymes were measured using 96-well microplates (Greiner) with 8 mU/mL of DPP IV enzyme and 200 μM of substrate (Gly-Pro-AMC). Changes in fluorescence were measured with an excitation (370 nm) and emission (440 nm) with 2.5 nm slit width by Flex Station 3 (Molecular Devices, CA, USA). Detailed methodology is given in (Materials and Methods section: S1.5 in S1 File).

### Starch digestion

The effects of HWHF on starch digestion were measured as described in detail elsewhere based on sequential incubations of starch with heat stable α-amylase and amyloglucosidase (Sigma-Aldrich, St. Louis, USA) [20]. Starch (100 mg) was suspended in water in the presence/absence of HWHF. A heat stable α-amylase (0·01%) (Sigma-Aldich) was added to the mixture and incubated for 20 min at 80 °C. After incubation, the reaction was further treated with 0·1% of Rhizopus mold amyloglucosidase (Sigma-Aldich) for 30 min at 60 °C. Samples were aliquoted and stored for subsequent analysis of glucose using the GOD/PAP method (Randox GL 2623). Acarbose was used as a positive control.

### Glucose diffusion *in vitro*

A simple *in vitro* method was used to assess the effects of HWHF on endogenous glucose diffusion [26]. The model comprised cellulose ester (CE) dialysis tubes (20 cm × 7.5 mm, Spectra/Por^®^CE layer, MWCO: 2000, Spectrum, Netherland) loaded with 2 ml 0.9% NaCl supplemented with 220 mM glucose containing HWHF. The two ends of each tube were securely sealed and put in 45 ml 0·9% NaCl. The tubes were kept on an orbital shaker with the temperature maintained at 37 °C for 24 h. Glucose diffused into the exterior solution was estimated using the GOD/PAP method (Randox GL 2623) with 0.5 ml aliquots of dialysate mixture.

### Animals

Sprague-Dawley male rats from Envigo UK (approximately 350–400 g) fed for 5–6 weeks on a high-fat diet (20% protein, 45% fat and 35% carbohydrate; 26.15 KJ/g total energy percent, Special Diet Service, Essex, UK) were used. Rats fed standard rodent diet (10% fat, 30% protein and 60% carbohydrate, 12.99 KJ/g total energy, Trouw Nutrition, Cheshire, UK) were used as controls. Other experiments examining effects of quercitrin on oral glucose tolerance were performed using 6–8 weeks old, male Swiss albino mice (Envigo) maintained on same standard diet. A final series of experiments evaluated the effects of HWHF on residual gut sucrose content, intestinal glucose absorption and disaccharidase activity together with gastrointestinal motility *in vivo*. Rats were anesthetized with an intraperitoneal injection of sodium pentobarbital solution (50 g/kg) and a midline incision of the abdomen was made from approximately 1 cm below the xiphoid to the pelvis. The intestine was resected into 5 segments: the upper 20 cm, middle, and lower 20 cm of the small intestine, the cecum, and the large intestine.

### Ethical approval

All studies were approved by the Animal Welfare and Ethical Review Board (AWERB) at Ulster University and conducted in accordance with UK Animals (Scientific Procedures) Act 1986 and EU Directive 2010/63EU. All necessary steps were taken to prevent any potential animal suffering.

### Oral glucose tolerance

The oral glucose tolerance tests were carried out using high-fat-fed (HFF) rats to assess effects of HWHF and its identified component, quercitrin on glycaemic control. HFF rats were fasted overnight and administered with glucose (18 mmol/kg, body weight (b.w.)) alone (control) or in combination with HWHF (250 mg/5ml/kg, b.w.) and quercitrin (30 mg/5ml/kg). Tail vein bleeding was carried out and blood glucose levels were measured at 0 min (prior to oral administration) and at 30, 60, 120, 180, 240, 360, and 480 mins afterwards using an Ascencia Contour Blood Glucose Meter (Bayer, Newbury, UK). Blood samples were collected in heparinized microvessel blood collection tubes (Sarstedt, Numbrecht, Germany). Plasma was separated from the blood via centrifugation at 12000 rpm for 5 min at 4 °C and stored at -20 °C for insulin measurement by radioimmunoassay as described previously [27].

### DPP-IV enzyme activity *in vivo*

Plasma DPP-IV activity were evaluated by fluorometric analysis on the basis of release of AMC (7-Amino-4-Methyl-Coumarin) from Gly-Pro-AMC substrate by amendment of the technique as reported before [25]. HFF rats were fasted overnight, and blood samples collected at different time points as mentioned in Fig 3B (0, 30, 60, 120, 180, 240, 360, and 480 mins) following oral gavage of HWHF (250 mg/5ml/kg, b.w.), sitagliptin (10 μmol/5ml/kg, b.w.), valdagliptin (10 μmol/5ml/kg, b.w.) or saline control. Plasma samples (10 μl) were incubated at 37 °C for 30 min with 40 μl of Tris-HCl (100 mM) buffer (pH 7.4) and 50 μl of Gly-Pro-AMC (200 μM) substrate in each well of 96 well microplates. The fluorescence product, 7-Amino-4-Methyl Coumarin (AMC), was released when the blood serum containing DPP-IV enzyme hydrolysed the fluorogenic substrate bonds (H-Gly-Pro) conjugated to the AMC group (H-Gly-Pro-AMC). Fluorescence changes were measured with Flex Station 3 as described above in the section on DPP-IV enzyme activity *in vitro*.

### Residual gut sucrose content

Residual gut sucrose content was measured to determine the changes in sucrose absorption from the GIT after an oral sucrose load as recently reported [22]. The process was carried out by administering oral sucrose load (2·5 g/5ml/kg, b.w.) to 24h fasted HFF rats together with/without HWHF (250 mg/5ml/kg, b.w.). The rats were culled at different time points (0, 30, 60, 120 & 240 min) to monitor the residual sucrose content in the intestine. The GI tract was cut into six different fragments including the stomach, the upper (20 cm), middle and lower (20 cm) of the small intestine, the caecum, and the large intestine. After being cleansed with acidified ice-cold saline, the fragments were centrifuged for 10 min at 3000 rpm. The resulting supernatant was first boiled for 2 h with sulphuric acid to hydrolyze the sucrose content and then it was neutralized to pH between 7.0–7.4 by addition of NaOH (1 M). Glucose released from gastrointestinal residual sucrose was determined using the GOD/PAP method (Randox GL 2623).

### Intestinal glucose absorption

An intestinal perfusion technique *in situ* [28], was used to determine the impact of HWHF on intestinal glucose absorption in 36 h fasted HFF rats anaesthetized with sodium pentobarbital (50 g/kg, b.w.). HWHF (5 mg/ml equal to 0.25 g/5ml/kg, b.w.) was added to KRB buffer containing glucose (54 g/l). The resulting mixture was infused through the pylorus, and the perfusate was collected at the end of the ileum via a catheter. The control group was treated with KRB buffer only containing glucose. The rate (0·5 ml/min) and temperature (37 °C) of the perfusion was kept constant for 30 min. The amount of glucose in solution prior to and after the perfusion of intestine were determined using the GOD/PAP method (Randox GL 2623) and the effect of HWHF expressed as percentage of glucose absorbed.

### Intestinal disaccharidase activity and gastrointestinal motility

The activity of the disaccharidase enzyme in the gut was measured as previously reported [29]. Intestinal disaccharidase activity was assessed after oral administration of HWHF (250 mg/5ml/kg) to 24 h fasted HHF rats. A second group of HFF rats given an oral dose of saline only served as control. The rats were culled after 1 h and their small intestines were excised, segmented longitudinally, and cleansed with ice cold saline (0.9% NaCl). Tissue homogenization was performed immediately following the dilution with 10ml saline. Homogenate aliquots were incubated at 37 °C in a 40 mM sucrose solution for an hour. The disaccharidase enzyme inhibitor, Acarbose (200 mg/5ml/kg, b.w.), was used as a positive control. Disaccharidase enzyme activity was expressed as μmol/mg protein per hr.

Gastrointestinal motility was assessed using a solution of BaSO4 milk as reported earlier [22, 29]. HFF rats starved for 12 h were given HWHF (250 mg/5ml/kg, b.w.) orally 60 mins prior to oral administration of 10% BaSO4 (10% BaSO4 and 0·5% carboxymethyl cellulose; w/v) solution. The control group was treated with only distilled water (10 ml/kg). After 15mins, rats from both the groups were culled. The length of the small intestine travelled by the BaSO4 was measured and calculated as a percentage of a total length (from the pylorus to ileocecal junction). The established drug, Bisacodyl (1 mg/5ml/kg, b.w.) was used as positive control.

### Fractionation of a crude extract

Crude bark extract (HWHF) reconstituted in 0.12% (v/v; TFA/water) and analysed by RP-HPLC. Filtered extract was injected into a (22 x 250 mm) Vydac 218TP1022 preparative stainless steel 10 μm C-18 column (Grace, Deerfield, IL, USA), equilibrated with 0.12% (v/v; TFA/water) at the flow rate of 5 ml/min. Acetonitrile was used as eluent at linear gradients to 20% over 10min and to 70% over a period of 40 min. Peak fractions detected at 254 nm and 360 nm were collected and individual peak retention time noted [20]. Major peaks were tested for insulinotropic activity as per aforementioned methods, and positive peak fractions were analysed further using Vydac 208TP510 (10 x 250 mm) semi-preparative stainless steel 5μm C-18 column (Phenomenex, UK) at a flow rate of 1 ml/min.

### Mass spectroscopy

Molecular weight of peak samples of HWHF were determined using LC-MS via ESI-MS. The peak fractions were separated on a Spectra System LC (Thermo Separation Products) using a Kinetex 5 μm F5 LC column ((150 x 4.6 mm) (Phenomenex)) with UV detection at 220–360 nm, and system conditions were described previously [30]. Detailed methodology is given in (Materials and Method section: S1.6 in S1 File).

### Structural elucidation of isolated molecules

The identity of compound/s from HWHF was determined by HPLC, LC-MS and NMR. NMR spectra for P-2 fraction were recorded using a 600 MHz Bruker AVIII HD spectrometer outfitted with a 5 mm BBO H&F cryogenic test. Standard one-dimensional composite pulse sequencing (zgcppr) was used to obtain ^1^H NMR spectra with the accompanying instrumental settings: number of scans = 16; temperature = 298 K; relaxation delay = 16 s; pulse width = 11.5 μs; acquisition time = 1.7039 s; receiver gain = 28; spectral width = 9615.4 Hz; offset = 4125 Hz. ^13^C NMR spectra were obtained with the aid of the utilization of the reverse gated-decoupling pulse sequence (zgig) and the purchase parameters were set as follows: number of scans = 32; temperature = 298 K; unwinding delay = 10 s; pulse width = 6.8250 μs; procurement time = 3.9716 s; spectra width = 36,057.7 Hz; offset = 4125 Hz [31]. All spectra were physically staged and routinely baseline corrected. Spin-lattice rest time (T1) estimations of protons in glycerol and maleic acid were estimated using a classical inversion recovery pulse sequence with 10 relaxation delays (τ) extending from 0.01 to 20 s [31].

### Statistical analysis

Graph Pad prism 5, was used for all analysis and interpretation of data. Data were analysed by unpaired Student’s t-test (nonparametric, with two-tailed P values) and one-way ANOVA with Bonferroni post hoc tests. Values were expressed as Mean±SEM with significance denoted by P<0.05.

## Results

### Concentration-dependent effects of *H*. *fomes* bark on insulin release from BRIN-BD11 cells and isolated mouse islets

**Fig 1:**
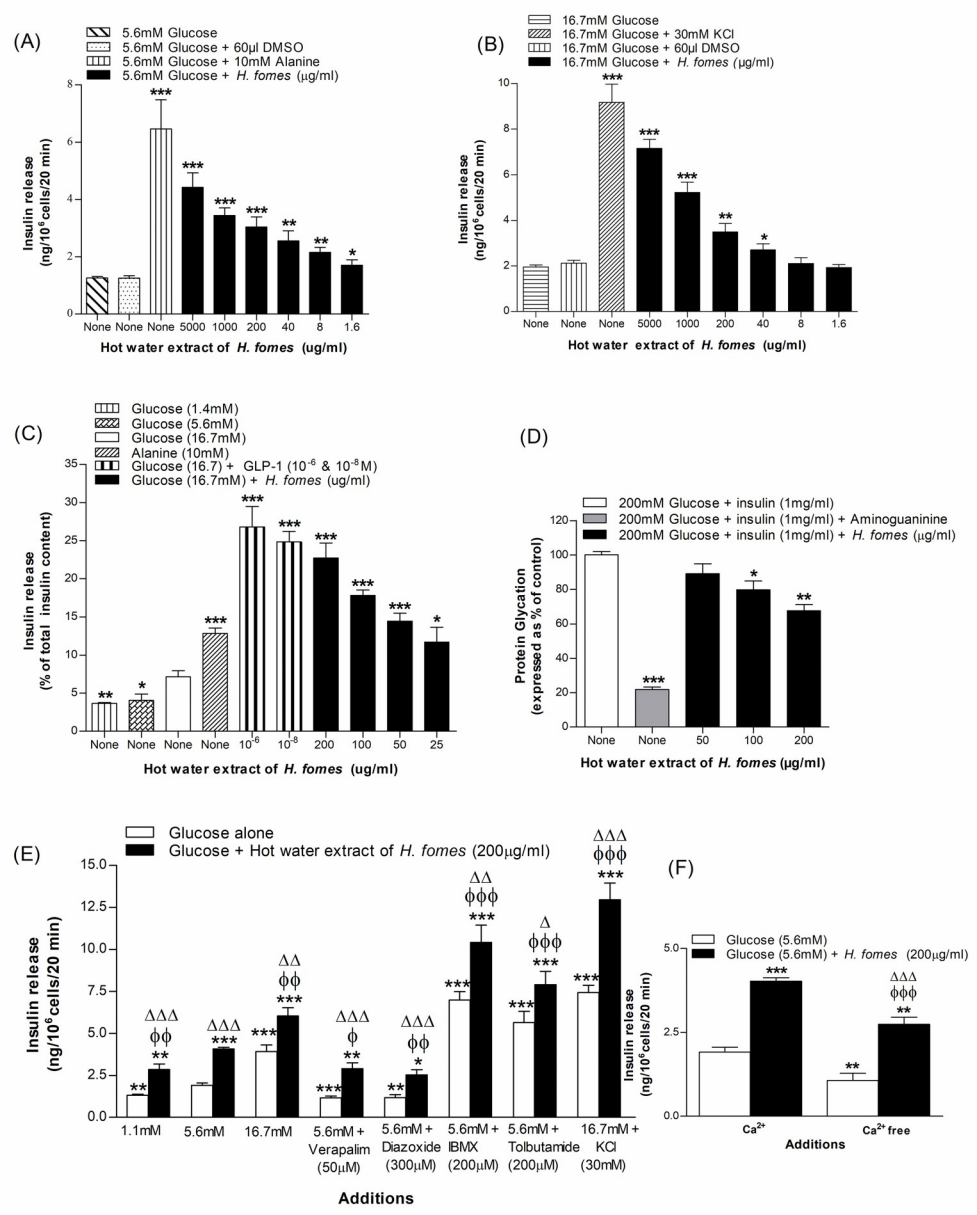
Effects of HWHF on insulin secretion from (A & B) BRIN-BD11 cells and (C) pancreatic islets, (D) protein glycation, (E) insulin release in the presence of established stimulators or inhibitors and (F) absence of extracellular calcium. Values are Mean±SEM for n = 4–8 for insulin secretion and n = 3 for protein glycation. *p<0.05, **p<0.01 and ***p<0.001 compared to control (5.6/16.7 mM glucose and 220 mM glucose + insulin (1 mg/ml)). ^ϕ^p<0.05, ^ϕϕ^p<0.01 and ^ϕϕϕ^p<0.001 compared to 5.6 mM glucose in the presence of HWHF. ^Δ^p<0.05, ^ΔΔ^p<0.01 and ^ΔΔΔ^p<0.001 compared to incubations without HWHF.

Basal insulin release from BRIN-BD11 cells was 1.3 ± 0.04 ng/10^6^ cells/20 min at 5.6 mM glucose. Alanine (10 mM) enhanced insulin output to 6.5 ± 1.0 ng/10^6^ cells/20 min. HWHF stimulated insulin release significantly (P<0.05–0.001) at ≥1.6 μg/ml concentrations (Fig 1A). Similarly, at 16.7 mM glucose, insulin release was 1.95 ± 0.08 ng/10^6^ cells/20 min whereas in the presence of 30 mM KCl, it increased to 9.18 ± 0.79 ng/10^6^ cells/20 min (Fig 1B). Moreover, at 16.7 mM glucose, HWHF enhanced insulin release significantly (P<0.05– P<0.001) at concentrations ≥40 μg/ml (Fig 1B). HWHF evoked concentration-dependent increase of insulin release at concentrations of up to 1000 μg/ml, without harming the cell viability (S1A & S1B Fig in S1 File). HWHF also increased insulin secretion from isolated mouse islets in a concentration-dependent manner at 16.7 mM glucose (Fig 1C), exerting significant (P<0.05–0.001) effects at concentrations ≥25 μg/ml. GLP-1 and alanine, which were used as positive controls, similarly triggered an insulin response (Fig 1C).

### Insulinotropic effects of *H*. *fomes* bark in the presence of known modulators of insulin release

To evaluate the underlying mechanism of insulin release, the effects of non-toxic concentration (200 μg/ml) of HWHF were examined on insulin release in presence of established stimulators and inhibitors (Fig 1E). In the presence of a K^+^ channel activator, diazoxide (300 μM) and L-type voltage-dependent Ca^2+^ channels blocker, verapamil (50 μM), insulin release induced by HWHF was decreased by 28–38% (Fig 1E). Furthermore, HWHF increased insulin release by 1.7-fold in KCl (30 mM; Fig 1E) induced membrane depolarized cells and exhibited synergized insulin release in presence of isobutyl methylxanthine (IBMX; P<0.001; Fig 1E) and tolbutamide (P<0.001; Fig 1E). Insulin releasing activity of HWHF was reduced to 32% in absence of extracellular calcium (P<0.01; Fig 1F).

### Effects of *H*. *fomes* bark on membrane depolarization and intracellular calcium concertation in BRIN-BD11 cells

**Fig 2:**
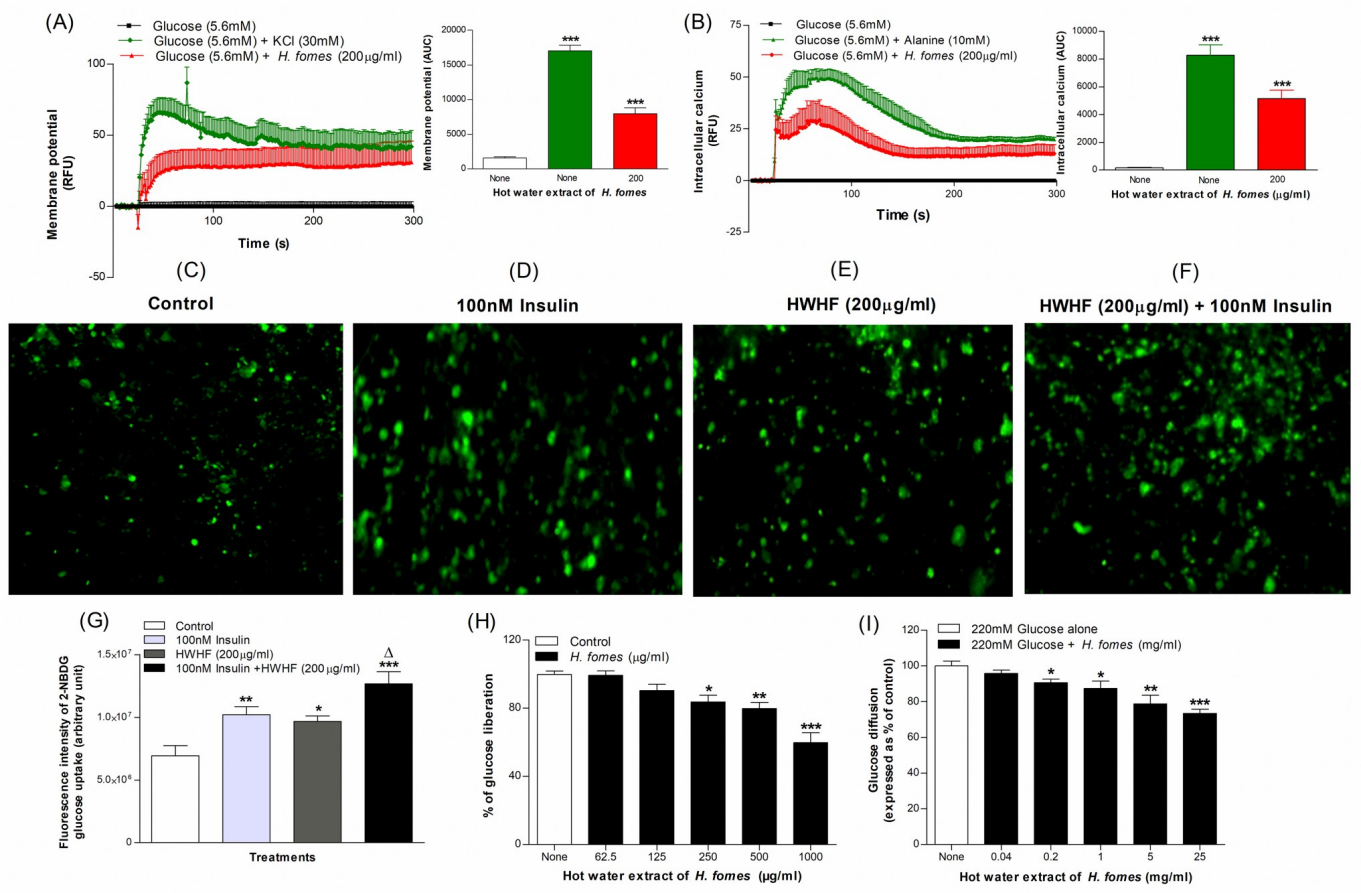
Effects of HWHF on (A) membrane potential and (B) cytoplasmic calcium in BRIN BD11 cells, (C, D, E, F & G) glucose uptake by differentiated 3T3L1 adipocytes, (H) starch digestion and (I) *in vitro* glucose diffusion. Fluorescence intensity was monitored in cells incubated with HWHF without (E) or with (F) 100 nM insulin. Images are taken at X10 magnification. Values are Mean±SEM for n = 6 for membrane potential and cytoplasmic calcium, n = 4 for glucose uptake, starch digestion and glucose diffusion. *p<0.05, **p<0.01 and ***p<0.001 compared to control.

HWHF induced significant (P<0.001) membrane depolarization and increased intracellular calcium by 56% to 80% (Fig 2A and 2B) respectively. The positive controls 30 mM KCl and 10 mM alanine respectively depolarised BRIN-BD11 cells by 91% and increased intracellular calcium by 98% (P<0.001; Fig 2A and 2B).

### Effects of *H*. *fomes* bark on glycation of insulin

A significant (P<0.05–0.01) inhibition in insulin glycation was observed with HWHF. At concentrations of 50–200 μg/ml, the extract caused 20–32% inhibition at ≥100 μg/ml concentration (P<0.05–0.01; Fig 1D). Aminoguanidine (44 mM) used as a positive control, caused 78% inhibition (P<0.001; Fig 1D).

### Effects of *H*. *fomes* bark on glucose uptake and insulin action

The effects of HWHF on uptake of fluorescent glucose in the presence or absence of insulin (100 nM) are shown in Fig 2(C)–2(F)). At 100 nM insulin, HWHF increased glucose uptake by 1.7-fold (P<0.05; Fig 2G). Insulin alone stimulated glucose uptake by 1.5- fold (Fig 2G) compared to basal control.

### Effects of *H*. *fomes* bark on starch digestion

HWHF significantly inhibited starch digestion at concentrations ≥250 μg/ml, with 40% inhibition (P<0.001) at 1000 μg/ml (Fig 2H). The positive control, acarbose (1 mg/ml) inhibited enzymatic glucose liberation by 87% (S1C Fig in S1 File).

### Effects of *H*. *fomes* bark on glucose diffusion *in vitro*

HWHF at 0.2–25 mg/ml resulted in significant (P<0.05–0.01) inhibition in glucose diffusion (Fig 2I). The extract caused 27% inhibition at 25 mg/ml (Fig 2I). Guar gum, a well-known intestinal glucose absorption inhibitor used as positive control, inhibited glucose diffusion by 51% at 25 mg/ml (S1D Fig in S1 File).

### Effects of *H*. *fomes* bark on DPP-IV enzyme activity *in vitro*

**Fig 3:**
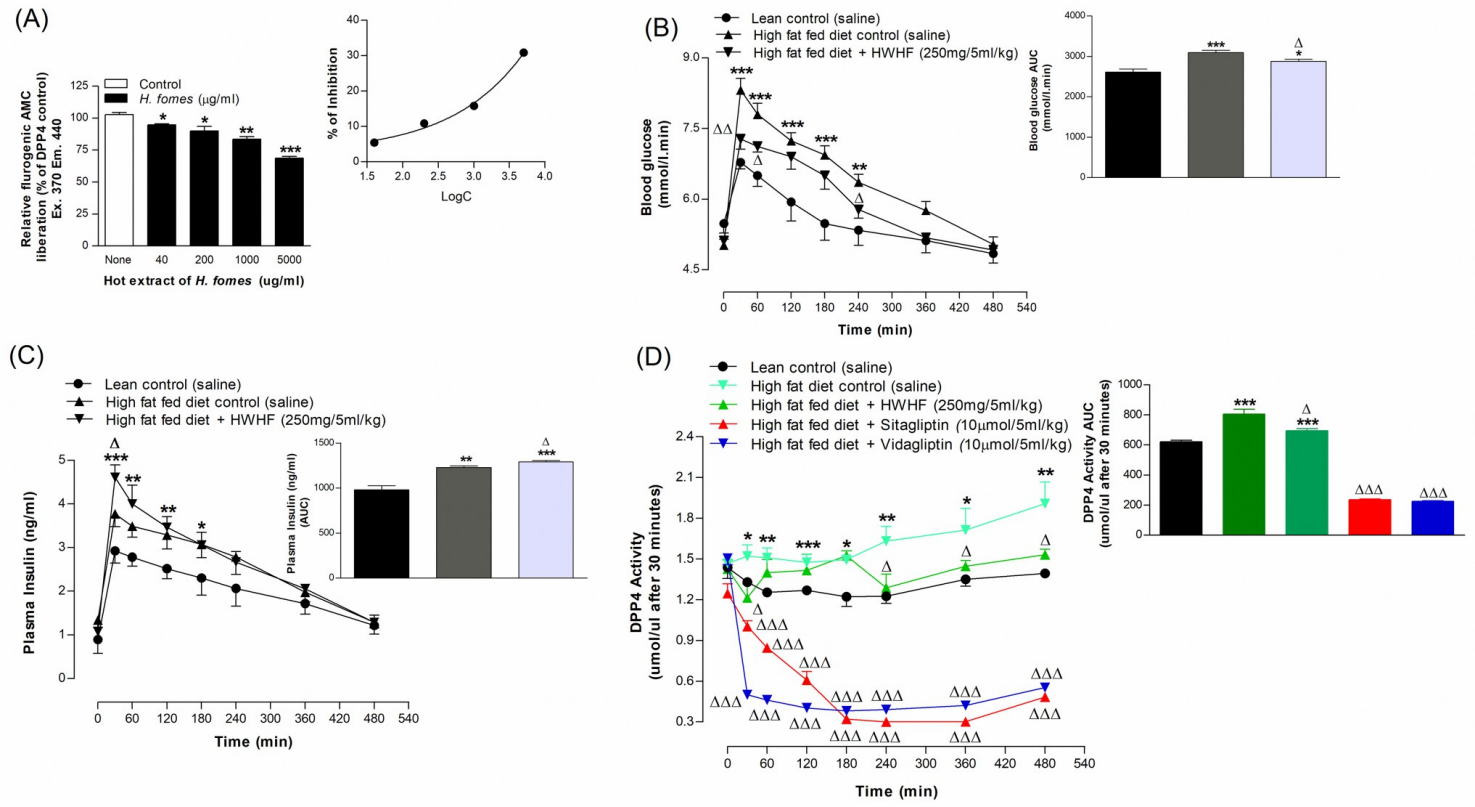
Acute effects of HWHF on (A) DPP-IV enzyme activity *in vitro*, (B) glucose tolerance, (C) plasma insulin and (D) plasma DPP-IV in high fat fed rats. Parameters were assessed before and after oral administration of 18 mmol glucose/kg, body weight (control) with or without HWHF (250 mg/5ml/kg, b.w.). Established DPP-IV inhibitors: sitagliptin and vildagliptin, were used as positive controls. Values are Mean±SEM, n = 4 for DPP-IV enzyme activity *in vitro* and n = 6 for glucose tolerance, plasma insulin and DPP-IV *in vivo*. *p<0.05, **p<0.01 and ***p<0.001, compared to normal control and ^Δ^p<0.05, ^ΔΔ^p<0.01 and ^ΔΔΔ^p<0.001 compared to control.

HWHF caused 5–31% inhibition of DPP-IV enzyme at 40–5000 μg/ml (P<0.05–0.001, Fig 3A). Sitagliptin (10 μM), an established DPP-IV enzyme inhibitor, reduced DPP-IV activity by 98% (S1E Fig in S1 File).

### Acute effects of *H*. *fomes* bark on oral glucose tolerance

HWHF at a dose of 250 mg/5ml/kg, body weight (b.w.) induced a significant (P<0.05–0.001) reduction in blood glucose of high fat fed rats at 30, 60 and 240 mins after oral glucose compared to saline controls (Fig 3B). *H*. *fomes* treatment also increased plasma insulin concentrations at 30 min (P<0.05; Fig 3C). Area under the curve analysis showed an overall 12% (P<0.05) decrease in blood glucose and 9% increase in plasma insulin respectively (P<0.05; Fig 3(B) & 3(C)).

### Acute effects of *H*. *fomes* bark on DPP-IV enzyme activity *in vivo*

HWHF (250 mg/5ml/kg, b.w.) induced significant (P<0.05) reduction in DPP-IV enzyme activity of high fat fed rats at 30, 240, 360 and 480 mins after treatment compared to saline-treated controls (Fig 3D). Area under the curve analysis showed an overall 14% (P<0.05) decrease in DPP-IV enzyme activity (Fig 3D). Sitagliptin and vildagliptin (10 μmol/5ml/kg, b.w.) used as positive controls induced 69–71% (P<0.001; Fig 3D) reductions in DPP-IV enzyme activity.

### Effects of *H*. *fomes* bark on gastrointestinal tract sucrose content after oral sucrose loading

**Fig 4:**
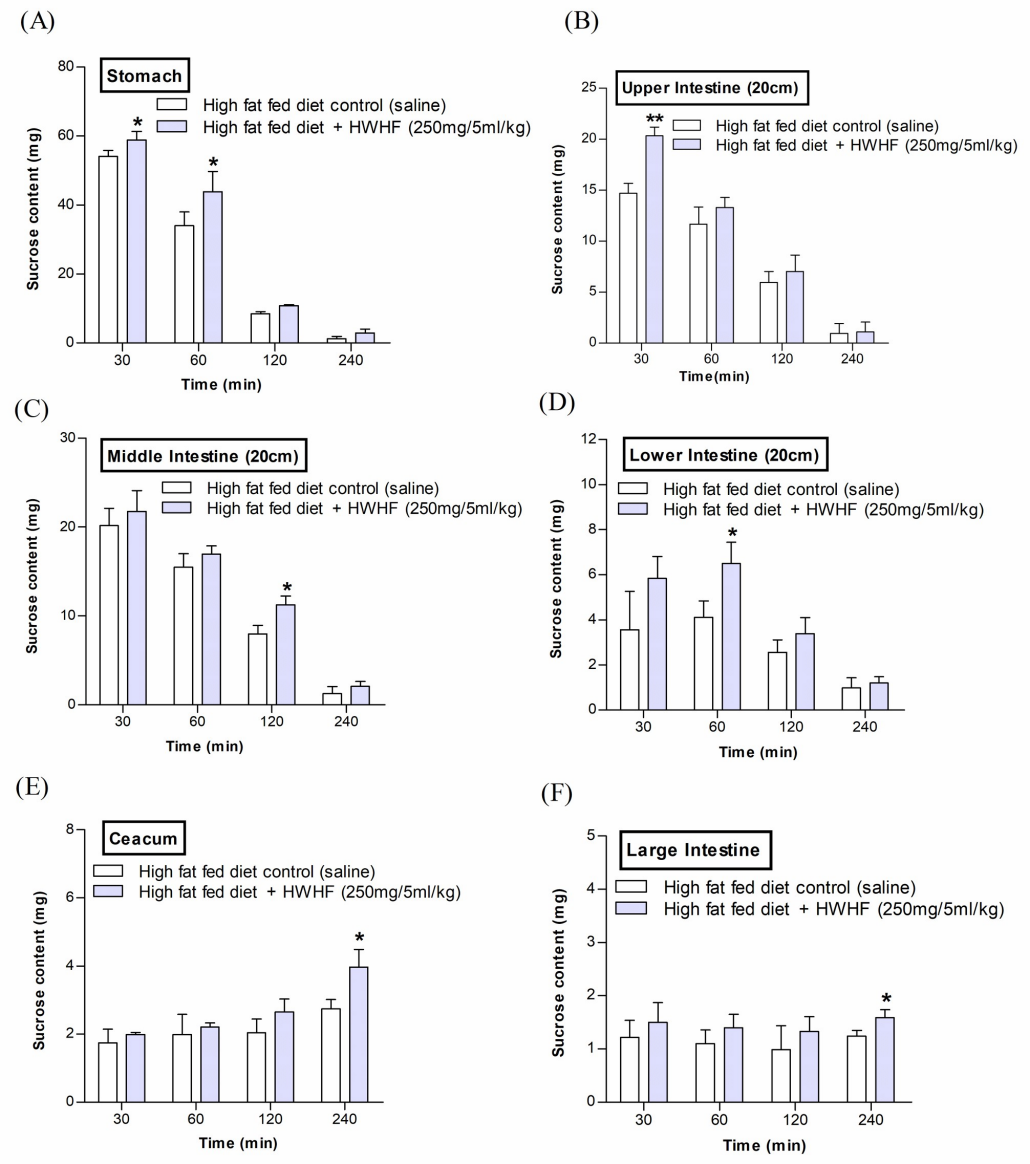
Effects of HWHF on (A-F) sucrose content in the gut after oral sucrose loading in high fat fed rats. Rats were fasted for 24 h before the oral administration of sucrose solution (2.5 g/kg b.w.) with or without HWHF (250 mg/5ml/kg, b.w.). Values are Mean ± SEM, n = 6. *p<0.05 and **p<0.01, compared to control.

A substantial amount of unabsorbed sucrose (P<0.05–0.01) was found in the GI tract (in the stomach and upper intestine at 30 and 60 min, and in middle and lower intestine at 60 and 120 min) following the oral sucrose gavage (2.5 g/5ml/kg, b.w.) with HWHF (250 mg/5ml/kg, b.w.) (Fig 4A–4D). Additionally, at 240min, a small amount of unabsorbed sucrose remained in the caecum and large intestine (P<0.05; Fig 4E and 4F). This implies rapid hydrolysis and absorption of sucrose in the upper GIT (Fig 4).

### Effects of *H*. *fomes* on intestinal glucose absorption

**Fig 5:**
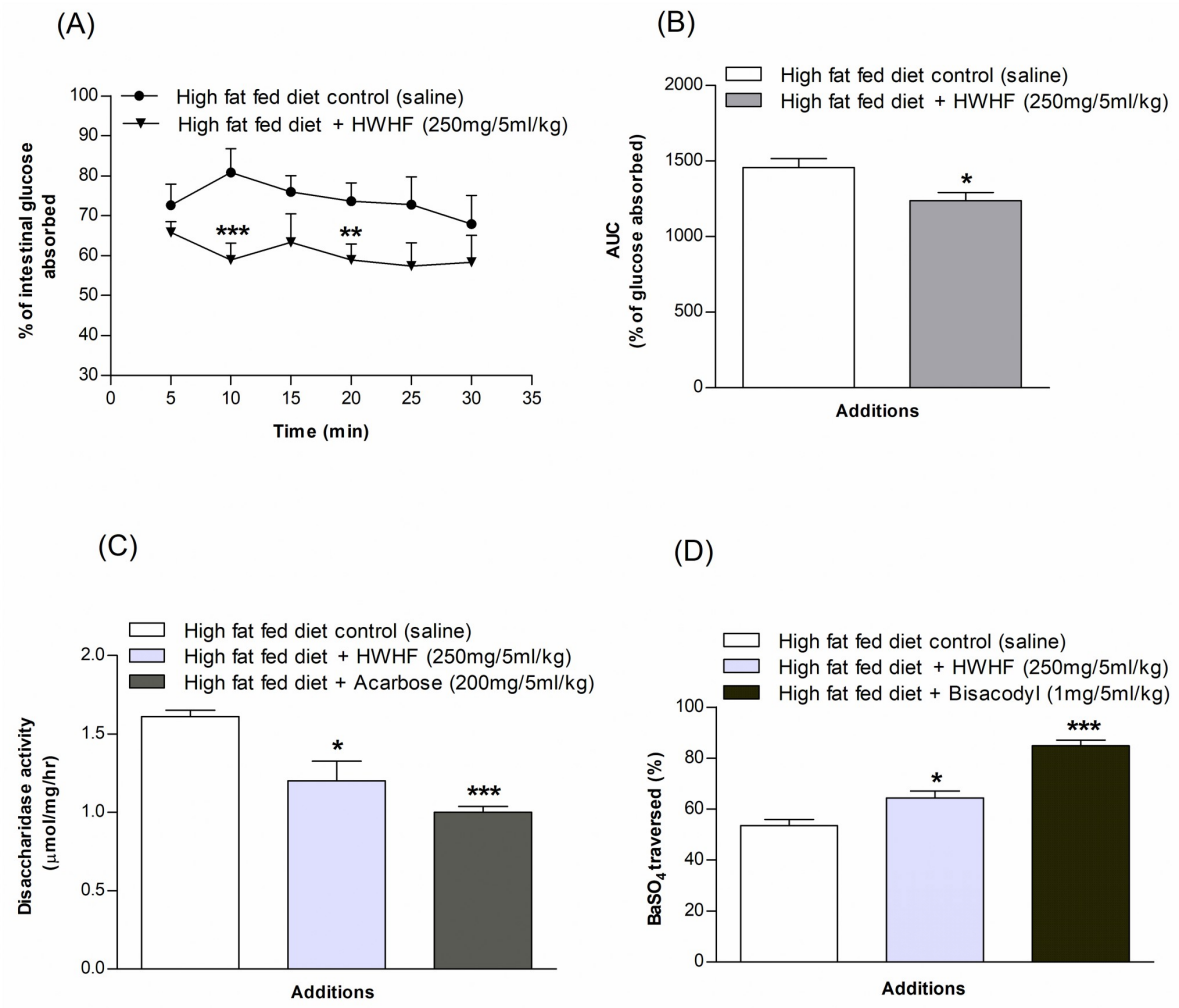
Effects of HWHF on (A & B) gut perfusion, (C) intestinal disaccharidase enzyme activity and, (D) GI motility in high fat fed rats. Rats were fasted for 36 h, and intestinal perfusion was performed with glucose (54 g/l) with or without HWHF (250 mg/5ml/kg, b.w.). BaSO4 was administered at 1 h following the initial oral dosing. Acarbose (200 mg/5ml/kg, b.w.) and Bisacodyl (1 mg/5ml/kg, b.w.) were used as positive controls for disaccharidase enzyme activity and GI motility, respectively. Values are Mean ± SEM, n = 8. *p<0.05, **p<0.01 and ***p<0.001, compared to control.

Fig 5A shows marked (P<0.01–0.001) diminution of glucose absorption rate in the gut after oral gavage of glucose with HWHF (250 mg/5ml/kg, b.w.). HWHF resulted in a substantial decrease in glucose absorption at 10 and 20 min (P<0.01–0.001). Area under the curve showed 15% decrease overall in glucose absorption (P<0.05; Fig 5B).

### Effects of *H*. *fomes* on intestinal disaccharidase enzyme activity and GI motility

HWHF (250 mg/5ml/kg, b.w.) was found to significantly (P<0.05; Fig 5C) inhibit disaccharidase enzyme action. Furthermore, a notable increase in gastrointestinal motility was also observed at same dose (P<0.05; Fig 5D) compared to controls. The established drugs, Acarbose (200 mg/5ml/kg, b.w.) and Bisacodyl (1 mg/5ml/kg, b.w.) respectively decreased disaccharidase enzyme activity (P<0.001; Fig 5C) and increased GI motility (P<0.001; Fig 5D).

### Structural elucidation of isolated molecules

**Fig 6:**
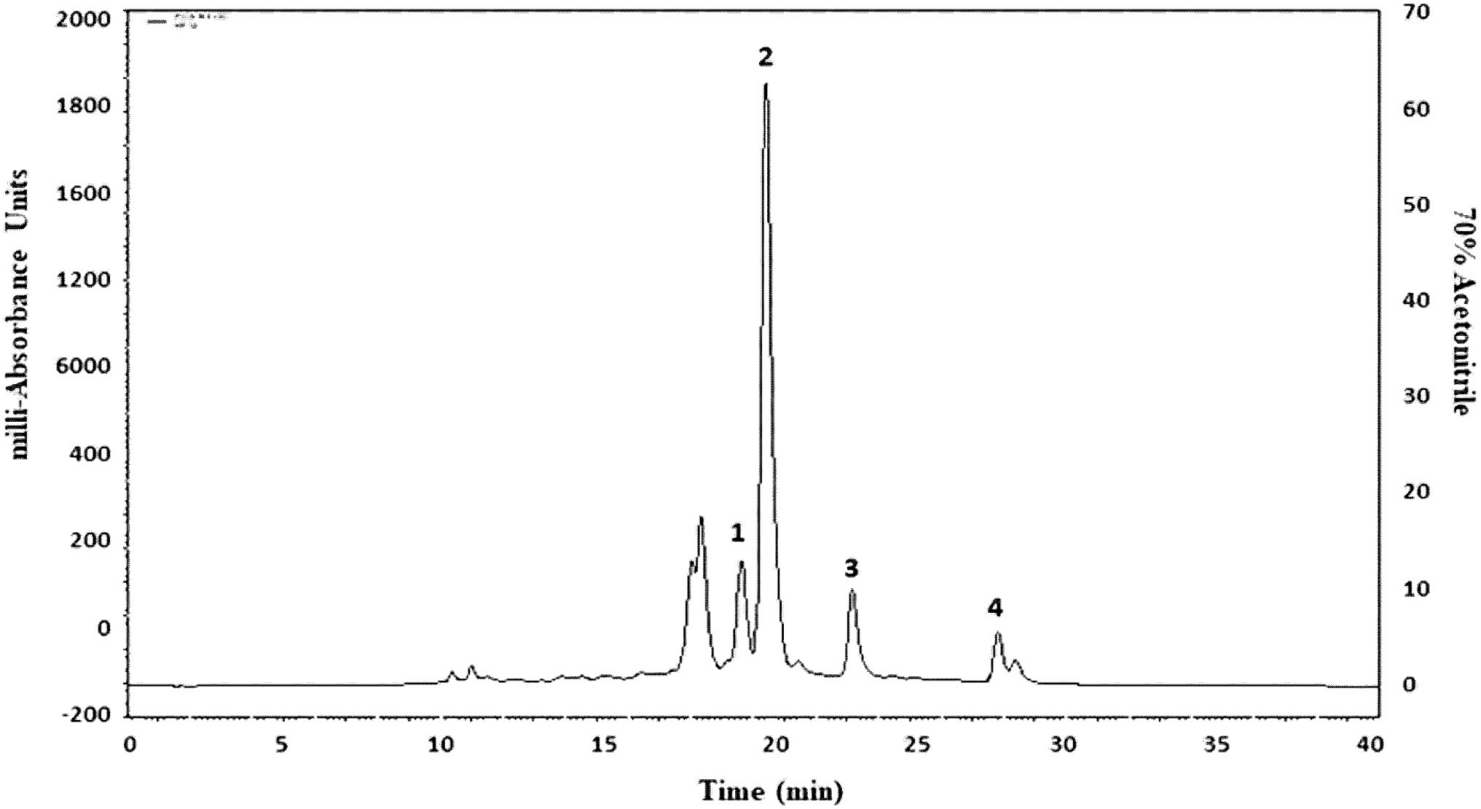
Representative HPLC profile of HWHF. Crude HWHF was chromatographed with flow rate of 1.0 ml/min on a (10 x 250 mm) semi-preparative 5μm C-18 column (Phenomenex, UK). The concentration of the eluting solvent was raised using linear gradients of acetonitrile (0–20% from 0 to 10 min, 20–70% from 10 to 40 min and 70–20% from 40 to 60 min). Compounds were detected by measurement of absorbance at 254-360nm.

**Fig 7:**
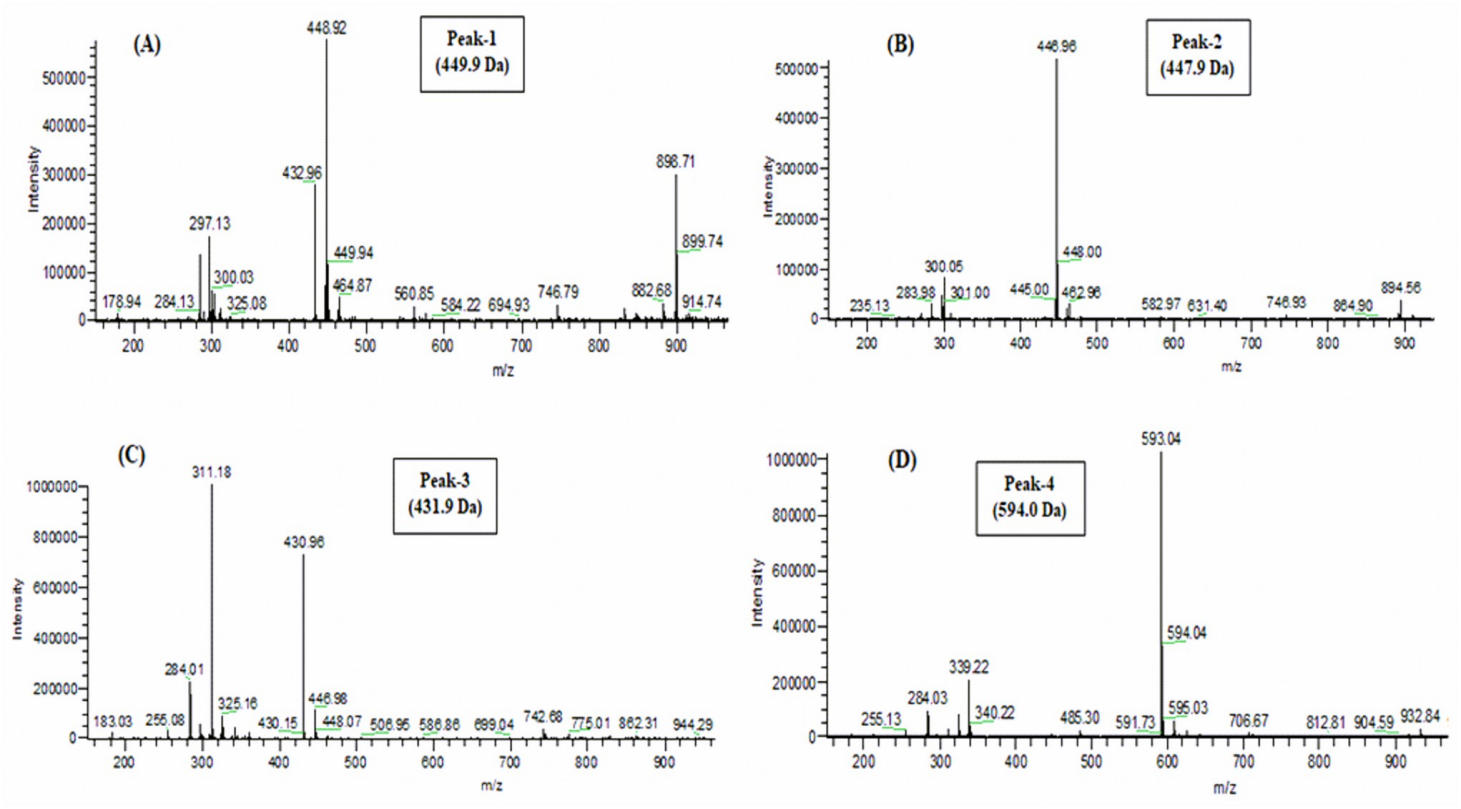
Molecular mass of peak samples of HWHF by LC-MS analysis. Peak fractions were separated on a Spectra System LC using a Kinetex 5μm F5 LC column (150 x 4.6 mm) (Phenomenex). The mass-to-charge ratio (m/z) versus peak intensity was determined. Samples of “peaks (P) 1 to 4” with retention times of 19, 20, 23 and 28 min were used to determine the molecular weights of unknown compounds with m/z 449.9, 447.9, 431.9 and 594.0 Da, respectively.

**Fig 8:**
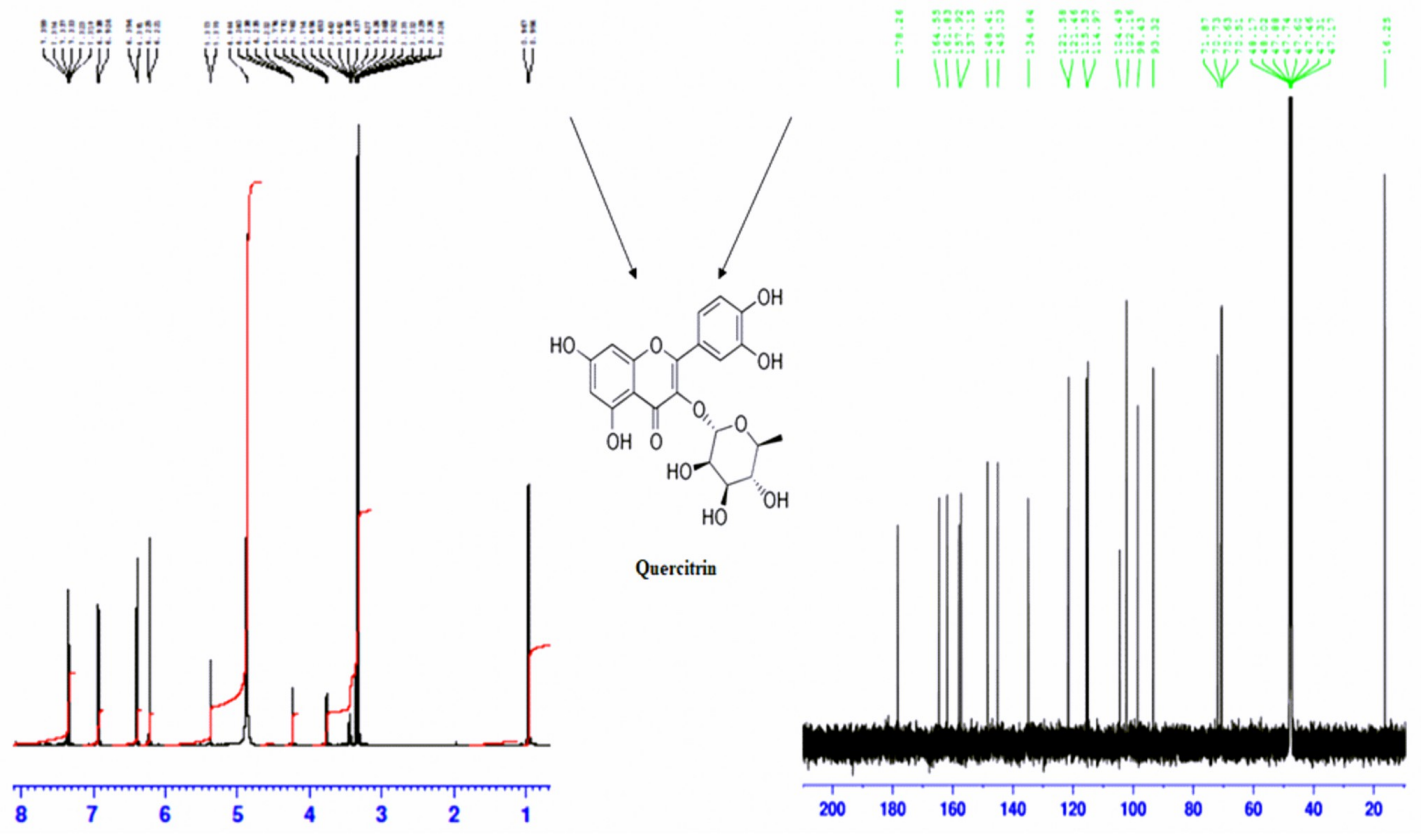
(A) ^1^H-NMR, (B) C^13^-NMR spectrum and isolated compounds (C) Quercitrin of P-2 sample obtained from RP-HPLC of HWHF. Proton-decoupled natural abundance ^1^H- NMR and C^13^- NMR spectrum of peak-2 sample of HWHF (obtained from chromatograph over the period of 70% acetonitrile from 10 to 40 min with retention time of 20 min) at 40 °C. The spectrum was obtained at 600 MHz after 119044 transients (14 h) by the pulsed Fourier transform method on a Varian XL-100 A spectrometer. Representative structure of flavonoids, corresponding to the molecular formula of quercitrin is C_21_H_20_O_11_.

**Fig 9:**
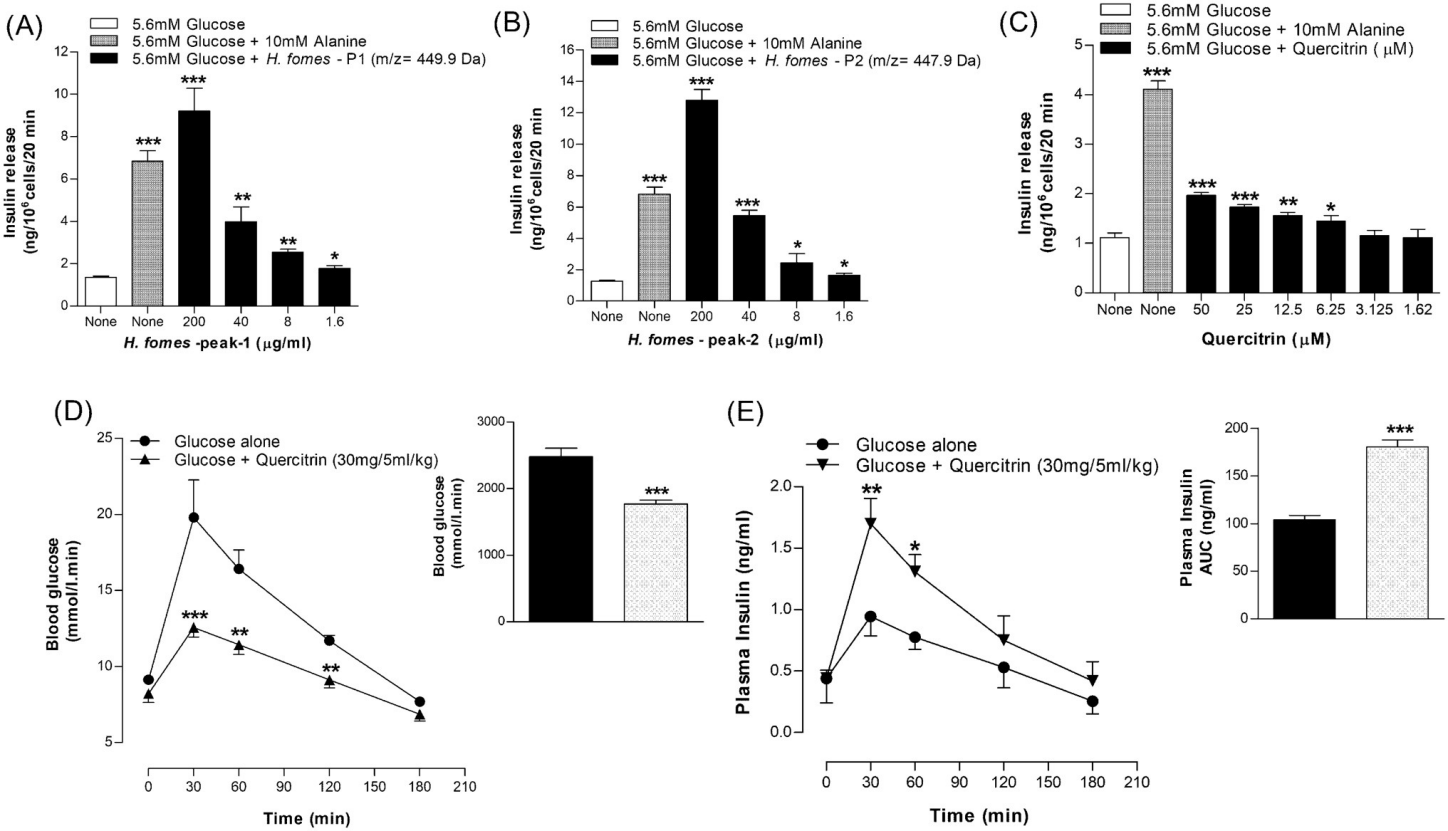
Insulin releasing effects of (A & B) Peak-1 & 2, (C) Quercitrin from BRIN-BD11 cells and, (D & E) glucose tolerance and plasma insulin in mice. Fasted (12 h) mice were administered glucose (18 mmol /5ml/kg, body weight) orally with or without (D & E) quercitrin (30 mg/5ml/kg b.w.). Values are Mean±SEM for n = 8 for insulin release and n = 7 for glucose tolerance and plasma insulin. *p<0.05, **p<0.01 and ***p<0.001 compared to control.

Phytochemicals in crude HWHF possibly responsible for observed bioactivity were purified by HPLC, used for molecular mass determination by LC-MS and structure elucidation by NMR (Figs 6–8). The molecular masses of P-1, P-2, P-3 and P-4 were 449.9, 447.9, 431.9 and 594 Da respectively (Fig 7(A)–7(D)). Evaluation of peak fractions using insulin-releasing bioactivity with BRIN-BD11 cells revealed that only P-1 and P-2 exhibited insulin secretory activity. As shown in Fig 9, P-1 & P-2 significantly stimulated (P<0.05–0.001) insulin release in a concentration-dependent manner (1.6–200 μg/ml) (Fig 9(A) & 9(B)). However, the high concentration (200 μg/ml) was associated with toxicity of cells (S1F & S1G Fig in S1 File). The LC-MS analysis of P-2 showed close similarity with quercitrin [32–34].

Unknown compound (P-2) was analysed through ^1^H (Fig 8A) and ^13^C (Fig 8B) NMR for structural characterization. C_21_ H_20_ O_11_ was obtained as yellow powder, λmax = 256, 360 nm; EI-MS *m/z* 447.9 Da [M]; (600MHz, CD3OD, ^1^H-NMR (δ in ppm)); Ring A: 6.39 (d, 1H, *J* = 1.8Hz), 6.22 (d, 1H, *J* = 2.4Hz); Ring B: 7.35 (d, 1H, *J* = 1.8Hz), 7.32 (dd, 1H, *J* = 2.4Hz, *J* = 8.4Hz), 6.92 (d, 1H, *J* = 8.4Hz); and Ring C: 5.37 (d, 1H, *J* = 1.8Hz), 4.24–3.32 (sugar H), 0.96 (d, 3H, *J* = 6.6). ^13^C NMR (600MHz, CD3OD, δ in ppm): 157.1 (C-2), 134.8 (C-3), 178.2 (CO-4), 161.8 (C-5), 98.4 (C-6), 164.5 (C-7), 93.3 (C-8), 157.9 (C-9), 104.4 (C-10); 121.5 (C-1’), 114.9 (C-2’), 145 (C-3’), 148.4 (C-4’), 115.5 (C-5’), 121.4 (C-6’) and 102.1 (C-R), 71.8 (C-R), 70.7 (C-R), 70.6 (C-R), 70.5 (C-R), 16.2 (C-R) [The letter R symbolize the signals of rhamnose molecule]. These findings indicate that P-2 was quercitrin which is one of the bioactive phytoconstituents. The molecular structure of this compound is shown in Fig 8. We purified very limited quantities of quercitrin which precluded direct biological testing. However, quercitrin was readily obtainable from commercial sources.

### Acute effects of quercitrin on insulin release from BRIN-BD11 cells

Quercitrin was tested for insulin secretory effects on BRIN-BD11 cells using alanine (10 mM) as a positive control (Fig 9C). Quercitrin induced a significant stimulation in insulin secretion at concentrations of 6.25–50 μM (P<0.05–0.001, Fig 9C). At higher concentration of 50 μM, it increased LDH release indicative of adverse effect on cell viability (S1H Fig in S1 File).

### Acute effects of quercitrin on oral glucose tolerance

At dose 30 mg/5ml/kg, body weight quercitrin induced a significant (P<0.05–0.01) improvement in oral glucose tolerance (18 mmol/kg, b.w.) at 30, 60 and 120 min respectively (P<0.05–0.001; Fig 9D). Quercitrin also increased plasma insulin at 30 and 60 min (P<0.05–0.01; Fig 9E). Area under the curve showed 29% (P<0.001) decrease in blood glucose (Fig 9D) and 32% (P<0.001) increase in plasma insulin responses (Fig 9E).

## Discussion

*H*. *fomes* is a popular medicinal herb in traditional medicine and has been claimed to possess antidiabetic properties [13]. This mangrove plant has been reviewed recently for pharmacological activities including antioxidant, antinociceptive, and cardiovascular benefits [13]. However, detailed scientific validation of these claims and knowledge of the underlying mechanism of action are lacking [13]. The present study aimed to carefully examine the antidiabetic potential of HWHF extract using a series of *in vitro* and *in vivo* studies to elucidate the possible mechanisms through which it can affect blood glucose homeostasis.

To achieve this goal, we used both *in vitro* and *in vivo* models including acute insulin release studies using BRIN BD11 cells and isolated islets together with in *vitro* evaluations of starch digestion, insulin glycation, glucose diffusion, cellular glucose uptake and DPP-IV inhibitory activity.

Such evaluation showed that hot water extract of *H*. *fomes* (HWHF) concentration-dependently stimulated basal and glucose-induced insulin secretion from both clonal and primary beta-cells. The insulin secretory effect of non-toxic concentrations was retained in the presence of tolbutamide, a K_ATP_-channel blocker or depolarisation with KCl (30 mM). However, diazoxide, a K_ATP−_channel opener [35], inhibited the insulin-releasing effects of HWHF indicating that cellular action of HWHF may involve closure of such channels as well as K_ATP_-independent effects. Consistent with this, verapamil, L-type voltage-dependent Ca^2+^ channel blocker, decreased HWHF activity, endorsing the importance of Ca^2+^- channel which was further validated by the effects of HWHF on membrane depolarisation and intracellular Ca^2+^ in clonal beta-cells. The effects of the extract were further potentiated by the cAMP phosphodiesterase inhibitor, IBMX [36]. This significantly enhanced the insulin releasing activity of HWHF. Interestingly, potential anti-oxidative and anti-inflammatory activity of *H*. *fomes* [37] have been proposed to be mediated by the cAMP pathway.

Obesity, decreased GLUT4 translocation or impairments in signal transduction of muscle and other target cells can cause cellular insulin resistance so agents that promote glucose uptake are desirable as antidiabetic drugs [38, 39]. HWHF significantly increased adipocyte glucose uptake and, although further detailed studies are required, this is assumed to be mediated via AMPK pathway [40]. This positive effect was observed in the presence and absence of insulin. Previous studies with naringenin, a flavonone, demonstrated insulin-stimulated glucose uptake in 3T3L1 adipocytes through inhibition of phosphoinositide 3-kinase (PI3K), a key regulator of insulin-induced GLUT4 translocation [41]. Therefore, it can be speculated that the increase in glucose uptake by HWHF might be due to the presence of flavonoids [42].

In the pathophysiology of diabetes, non-enzymatic glycosylation of structural proteins is considered as a major contributor to the onset of complications of the disease. Functional proteins such as insulin can also be glycated and this has been shown to decrease biological activity [43, 44]. In this study HWHF significantly decreased insulin glycation in a concentration-dependent manner. This might translate to improvements of insulin action *in vivo* but perhaps more importantly indicate that *H*. *fomes* might help protect structural proteins from the longer-term complications of diabetes. Interestingly, *H*. *fomes* has been reported to possess polyphenolic constituents such as flavonoids, that exhibit potential antioxidant properties [13]. Thus, this ability to inhibit glycation might be attributable to its antioxidative constituents [45].

The effects of HWHF on starch digestion were evaluated using Acarbose as positive control. The extract significantly inhibited starch digestion in a concentration-dependent manner, an action that may contribute to glucose lowering effects by slowing digestion and the passage of glucose into the circulation. *H*. *fomes* is rich in phytochemicals such as alkaloids and phenolic compounds [19] and previous studies reported that alkaloids and flavonoids exhibit α-glucosidase inhibitory activity [46]. Similarly, it has been reported that *H*. *fomes* bark contains a high content of fibre [47], that might additionally inhibit the nutrient absorption.

Many medicinal plants have been demonstrated to reduce gastrointestinal glucose absorption, that might contribute to their antihyperglyceamic properties [48]. In this study, we assessed the effects of *H*. *fomes* on glucose diffusion by using a simple dialysis-based *in vitro* method [49]. This model serves as a good proxy to mimic gastrointestinal absorption [26]. HWHF decreased glucose movement through the dialysis membrane in concentration-dependent manner. Guar gum, a well-known intestinal glucose absorption inhibitor, significantly inhibited the glucose diffusion. We therefore assume that this effect will contribute to decreased in glucose passage across the intestine and contribute to positive glucose-lowering activity.

Obesity and T2DM progression are interconnected. Many previous studies have reported that rats fed with high-fat diet develop obesity and associated metabolic disorders such as insulin resistance that may lead to diabetes [50]. In our acute *in vivo* study, oral administration of *H*. *fomes* significantly improved glucose tolerance and the plasma insulin to glucose in high-fat fed rats. These findings accord with the traditional use of HWHF for assisting diabetes control and are in agreement with a previous study that claimed significant anti-hyperglycaemic activity of *H*. *fomes* in streptozotocin-induced mice [14].

Dipeptidyl peptidase IV (DPP-IV) metabolises incretin hormones by N-terminal cleavage and liberation of GLP-1 (9–36) and GIP (3–42). This step serves to terminate the insulin-releasing and blood glucose lowering actions of the two incretin hormones [25]. As a result, inhibition of DPP-IV enzyme activity is now used clinically to enhance endogenous incretin action and treat type 2 diabetes [51]. The two peptides also inhibit glucagon secretion which is beneficial for blood glucose control [52]. In the present study, HWHF significantly inhibited DPP-IV enzyme activity *in vitro* and acutely *in vivo* when administered to high-fat fed rats. Previous studies reported that polyphenolic compounds such as flavonoids and flavanols glycosides exhibit DPP-IV enzyme inhibitory activity [27, 53]. Therefore, such phytochemicals in *H*. *fomes* may be responsible for this beneficial effect.

In an extension of these *in* vivo studies, HWHF noticeably decreased the absorption of glucose during intestinal perfusion with considerable amounts of unabsorbed sucrose persisting in all areas of the GIT. As suggested by *in vitro* studies, this indicates that HWHF restrains both carbohydrate digestion and absorption of glucose from the gut. Likewise, in a BaSO4 milk study, HWHF increased gut motility and reduced intestinal disaccharidase enzyme activity. These combined effects may limit the time available for absorption of carbohydrates [29] and therefore contribute to the improvement in the management of post-prandial hyperglycaemia. Part of these actions are potentially mediated through the formation of glucose-fibre complexes. *H*. *fomes* is known to contain polyphenols including proanthocyanidins which is good source of fibre [13]. However, it is important to note that dietary fibre has several influences on gastrointestinal tract, also including changes in transit time and the viscosity of the contents of the GI tract. These actions also influence food movements along the intestine, the enzymatic breakdown of food and interaction of nutrients with absorbing mucosal surface.

Although we could not attempt to isolate the various agents responsible for each the beneficial actions observed, we were able to evaluate the phytochemicals possibly contributing to the positive insulin secretory effects. The peak fractions (P-1 & P-2) collected from RP-HPLC of crude HWHF stimulated insulin release in a concentration-dependent manner.

Peak-2 sample of HWHF was further evaluated for identification and characterization of possible active compound using NMR. The ^1^H and ^13^C spectra showed signals of P-2 fraction. The ^1^H NMR spectrum exhibited two doublets at δ 6.22 (2.4 Hz) and δ 6.39 (1.8 Hz) of A ring. The presence of two doublets at δ 7.35 (1.8 Hz) and δ 6.92 (8.4 Hz) and one double doublet at δ 7.32 (2.4 & 8.4 Hz) were ascribed to B ring, respectively. The presence of two doublets at δ 5.37 (1.8 Hz) and δ 0.96 (6.6 Hz) showed in α substituted rhamnose ring. The ^13^C NMR spectrum showed resonance for 21 carbons including oxygenated aromatic carbons at δ 134.8 (C3), 161.8 (C-5), 164.5 (C-7), 145 (C-3’), 148.4 (C-4’) and 178.2 (CO-4). Thus, the spectral data obtained were identical to quercitrin as previously described in the literature [32–34]. The structural elucidation of the isolated compound P-2 revealed conclusively that it was quercitrin.

Further, we tested the effects of commercially available quercitrin on *in vitro* insulin release and glucose tolerance. The effects observed were indeed very similar to those observed with our isolated compound P-2 from HWHF, eliciting concentration-dependent insulin release from BRIN BD11 cells and improving glucose tolerance and plasma insulin responses in mice. Such findings are in agreement with previous studies that reported phenolic or polyphenolic compounds such as flavonoids enhance insulin secretion [10]. Although, this study used a good range of *in vitro* and *in vivo* methods to assess pharmacology of HWHF, our observations are limited by uncertainty regarding the exact molecular mechanisms involved and the precise identity of all of the active compounds responsible for such a wide range of antidiabetic actions.

## Conclusion

In summary, present study establishes that *H*. *fomes* extract possesses significant antihyperglycaemic properties mediated by multiple actions including the stimulation of insulin secretion, inhibition of starch digestion, disruption of glucose absorption and inhibition of DPP-IV which may enhance incretin action following feeding. Many populations in the world have limited access to modern antidiabetic drugs. The present data suggest that *H*. *fomes* and its phytoconstituents, such as quercitrin identified as compound P-2, may be useful as dietary adjuncts for the management of diabetes.

## Supporting information

S1 File (DOCX)
