# Supplementary material for: Insulin secretory and antidiabetic actions of Heritiera fomes bark together with isolation of active phytomolecules
Source: PLoS One. 2022 Mar 3;17(3):e0264632. doi: 10.1371/journal.pone.0264632 (PMC8893667; doi:10.1371/journal.pone.0264632)
Supplement: S1 File — (DOCX) [file pone.0264632.s001.docx]

**1. Materials and methods**

**1.1 Culture and maintenance of BRIN-BD11 Cells**

Clonal pancreatic β-cells (BRIN-BD11 cells) are an insulin-releasing cell line generated via electrofusion of RINm5f cells with New England Deaconess Hospital rat pancreatic islet cells. Cryogenic vials of 2ml (Greiner bio-one, UK) (1x10^6^ cells/vial) were used to store the cells in freezing medium composed of 10% (v/v) RPMI-1640 tissue culture medium (11.1 mM D-glucose), 80% (v/v) foetal bovine serum (FBS) and 10% (v/v) dimethyl sulfoxide (DMSO). Cells were gradually frozen from -20 °C for 4 hr and then moved to -70 °C overnight before transferring them to cryopreservation store. Cells were taken out from liquid nitrogen and defrosted in an ice bath around 20 min. RPMI-1640 medium was prepared with supplementation of 10% foetal bovine serum and 1% penicillin-streptomycin antibiotics (penicillin (100 U/ml), streptomycin (0.1 mg/l)) and then pre-warmed at 37 ^o^C around 10 min. The volume of 10 ml pre-warmed media was added gradually to prevent osmotic stress to the cells. The cell suspension was centrifuged at 900 rpm for 5 min (Universal 320, Hettich Zentrifugen, Germany). The supernatant was disposed of, and the cell pellet was resuspended in 15 ml pre-warmed RPMI-1640 medium and moved into a (75 cm^2^) sterile tissue culture flask (Nalge Nunc Universal, NY, USA). The cells were cultured at 37 ^o^C in an environment of 5% CO_2_ and 95% air (LEEC secure CO2 incubator, LEEC, Nottingham).

To subculture cells, Hank’s balanced salt solution (HBSS) of 10ml was used to wash the monolayer of cells which was attached to the surface of the flask. The volume of 3ml of pre-warmed trypsin (0.25% (w/v) containing 1 mM EDTA) was added to the flask and incubated at 37 ^0^C for 3-5 min to detach the cells and the detached cells were resuspended in to 10ml of RPMI-1640 medium and the cell suspension turned into centrifuged at 900 rpm for 5 min in pre-warmed RPMI-1640 medium of 20ml volume. 100 µl of cell suspension and 100 µl trypan blue was added in the Eppendorf to mix together and the Neubauer haemocytometer was used for counting the cells before seeding into the well plates for experiments. To maintain the cell propagation, 1 ml of cell suspension was resuspended into tissue culture flask and made up to 25 ml with pre-warmed RPMI-1640 medium containing 11.1 mM glucose and kept at 37 °C until the flask became around 80% confluent. Cells used in the test were between passages sixteen and forty.

**1.2 *In vitro* insulin-releasing studies using BRIN BD11 Cells**

Acute *in vitro* insulin release studies were investigated as described below. Trypsin/EDTA (0.25% (w/v)) were used to detached BRIN BD11 cells from the tissue culture flask and seeded in to sterile 24-well tissue culture plates at a density of 150,000 cells/well and incubated for 24 hr at 37 °C. After incubation the culture media was poured off and the cells were pre-incubated with 1 ml of Krebs Ringer Bicarbonate (KRB) buffer (115 mM NaCl, 4.7 mM KCl, 1.28 mM CaCl2, 1.2 mM MgSO4, 1.2 mM KH2PO4, 20 mM HEPES, 25 mM NaHCO3, 0.1% bovine serum albumin (BSA), pH 7.4) containing 1.1 mM glucose for 40 min at 37 °C. After pre-incubation, the assay buffer was replaced with 1 ml of treatments appropriate to each experiment. All the treatment groups were made in KRB buffer containing 5.6/16.7 mM glucose, unless otherwise mentioned. Treatment groups containing secretagogues, crude or purified plant extracts were added to their respective wells and incubated for 20 min at 37 °C. Aliquots (950 µl) were collected from each well and stored at -20 °C until the insulin radioimmunoassay was performed.

Insulin release modulators such as IBMX, tolbutamide, KCl, verapamil and diazoxide were used to study the mechanism of insulinotropic actions of plant extracts. BRIN-BD11 cells were incubated with KRB buffer containing 5.6 mM glucose and one of the modulators verapamil (50 µM), diazoxide (300 µM), IBMX (200 µM) or tolbutamide (200 µM)) in the presence or absence (control) of HWHF. Another set of experiments was carried out by incubating BRIN-BD11 cells with KRB buffer containing 16.7 mM glucose and KCl (30 mM) in the presence or absence (control) of HWHF. Insulin release studies in the absence of extracellular calcium were performed by incubating BRIN-BD11 cells in the same KRB buffer lacking added CaCl_2_ and supplemented with 0.1 mM EGTA plus 0.1% BSA (pH 7.4). The culture media was removed, and cells were incubated with 1 ml of calcium-free KRB buffer containing 1.1 mM glucose for 40 min at 37 °C. After pre-incubation, the cells were incubated with test treatments containing HWHF in calcium-free KRB buffer containing 5.6 mM glucose for 20 min at 37 °C.

**1.3 *In vitro* insulin-releasing studies using isolated mouse islets**

Adult mice were sacrificed following ethically approved and established protocols. Pancreatic islets were isolated using collagenase digestion. Hank’s Balanced Salt Solution ((HBSS), (8 g/l NaCl, 0.4 g/l KCl, 0.14 g/l CaCl2, 0.1 g/l MgCl2.7H2O, 0.1 g/l MgCl2.6H2O, 0.06 g/l Na2HPO4.H2O, 0.06 g/l KH2PO4, 1 g/l glucose, 0.02 g/l phenol red, 0.35 g/l NaHCO3, 10 mM HEPES and 5% BSA)) was used as wash buffer throughout the experiment. Collagenase-P solution (1.4 mg/ml), prepared in BSA-free HBSS, was injected into the pancreas. This caused the pancreas to inflate. The pancreas was then excised and kept in ice-cold wash buffer. The pancreas collagenase mix was placed in a shaking water bath at 37 ^o^C for 8-10 min. The tube was shaken vigorously 10-20 seconds to dissociate the tissue by mechanical digestion and placed in water bath for further digestion if mechanical digestion had not been achieved. Once islets were observed visually, the tube was immediately filled with ice cold wash buffer and centrifuged for 2 min at 1200 rpm. Supernatant was discarded and the wash step was repeated twice in succession. The solution was filtered through a strainer to remove undigested tissue and particles. The tube was then filled with wash buffer and centrifuged for 2 min at 1200 rpm. The supernatant was discarded, and the pellet was resuspended in 10 ml Histopaque (Sigma, Poole, UK) for density gradient separation. The tube was further centrifuged for 20 min at 1200 rpm and the supernatant containing the islets was collected into a tube with 25 ml of wash buffer. The tube was centrifuged at 1500 rpm for 4 min and the pellet was resuspended in wash buffer. The tube was further centrifuged at 1200 rpm for 3 min and the pellet was resuspended in RPMI media containing 10% BSA and 1% antibiotics and transferred to Petri dishes. Islets were cultured at 37 ^o^C and 5% CO_2_. After 48 hr culture in RPMI media, groups of ten islets were collected via observation under a microscope and added in 1.5 ml Eppendorf tubes. Isolated islets were pre-incubated with 500 μl of KRB buffer (115 mM NaCl, 4.7 mM KCl, 1.28 mM CaCl2, 1.2 mM MgSO4, 1.2 mM KH2PO4, 20 mM HEPES, 25 mM NaHCO3, 0.1% bovine serum albumin (BSA), pH 7.4) containing 1.4 mM glucose for 1 hr at 37 °C. After pre-incubation, test incubations with HWHF and insulin secretagogues had been carried out using KRB buffer containing 16.7 mM glucose for 1 hr at 37 ˚C. Aliquots of the produced supernatant were removed and stored at -20 ˚C for insulin radioimmunoassay.

Cellular insulin content of the islets was measured using acid-ethanol extraction method. The islets used for insulin release studies were treated with 500 μl of acid-ethanol solution (1.5% HCl, 75% ethanol and 23.5% H2O). The islets were disrupted using pipette tips and incubated at 4 ˚C overnight. Tubes containing the islets were centrifuged at 1200 rpm for 2 min and the supernatant was kept at -20 ˚C for insulin measurement using the insulin radioimmunoassay.

**1.4 Cellular Glucose Uptake**

3T3-L1 cells were acquired from the American Type Culture Collection ((ATCC), Virginia, USA). Dulbecco's modified eagle medium (DMEM) was prepared via supplementation of DMEM with penicillin (50 U/ml), streptomycin (50 µl/ml) and foetal bovine serum (10% v/v). 3T3-L1 fibroblasts cells were cultured, and adipocyte differentiation initiated for experimentation.

3T3-L1 differentiated cells were incubated with serum-free DMEM media for 2hr at 37 ^o^C, atmospheric at 5% CO_2_ in the incubator. After the incubation, the DMEM media was discarded. 200μl of Krebs-Ringer Bicarbonate buffer (KRBB) ((116 mM NaCl, 4.7 mM KCl, 1.28 mM KH2PO4, 1.2 mM MgSO4, 24 mM NaHCO3, 10 mM HEPES, (pH 7.4)) was added into each well and incubated for 30 min at 37 ^o^C incubator in an atmosphere of 5% CO_2_ and 95% air. To each experimental group of wells, 50µl of 200µg/ml concentration of treatment with or without 100nM insulin was added and incubated for 30min at 37 ^o^C. Finally, 2-NBDG, 2-(N-(7-Nitrobenz-2-oxa-1,3-diazol-4-yl) Amino)-2-Deoxyglucose (50nM) was added in each well incubated for 5 min. The wells were washed with ice-cold PBS (0.5-1ml) and a sufficient amount of normal PBS, or warm (37 ^o^C) PBS was added to cover the coverslip. Three to four coverslips were mounted in each slide and blocked with nail polish. Images of four corners of the coverslips using a 10X magnification on the microscope were captured. The fluorescence intensity was measured.

**1.5 DPP-IV enzyme activity**

An *in vitro* fluorometric method was implemented to determine the DPP-IV inhibitory activity of the EEHF. A Tris-HCl (100mM) buffer was prepared (0.2 M Tris-HCl and 0.1 M NaCl) and the pH was adjusted to 8.0 by using a 100mM Tris-base. Tris-HCl (100mM) was added to each well plate including the following 10 μl of a sample and 50 μl of 200 μM of substrate (Gly-Pro-AMC) and incubated for 5-10 min at 37 ^o^C. Finally, 10 μl of DPP-IV was added to initiate the reaction process after a 30 min incubation at 37 ^o^C. Any changes in fluorescence were observed using a FlexStation 3 (Molecular Devices, CA, and the USA) with excitation and emission at 370 nm and 440 nm with a 2.5 nm slit width. The observing parameter for this experiment was the rate of fluorescence emission by each sample tested. The inhibitory activity of dipeptidyl peptidase-IV was calculated by using the percent of inhibition by HWHF at the various concentrations.

**1.6 Mass Spectroscopy**

Molecular weight of peak samples were determined using Liquid Chromatography-Mass Spectrometry (LC-MS) via Electrospray ionization mass spectrometry (ESI-MS). Fractions (peak samples) were separated on a Spectra System LC (Thermo Separation Products) using a Kinetex 5µm F5 LC column ((150 x 4.6 mm) (Phenomenex)). A 10µL injection of the fractions was chromatographed at ambient temperature using gradient elution. The column was allowed to equilibrate for 4 min at 90% water: 10% acetonitrile, then increasing to 70% acetonitrile over 16 min and returning to the initial conditions for 5 min to allow re-equilibration before the next injection. A flow rate of 0.8 ml/min was maintained over the 25.10 min run time with UV detection at 220nm and 360 nm. The LC effluent was routed to an LCQ electrospray ion trap mass spectrometer (Thermo Finnigan, San Jose, CA, USA) for mass characterization. The system was controlled using Xcalibur software (Thermo Finnigan) and operated in negative ion mode, scanning over the mass range m/z 150.0 – 1200.0 Da. Nitrogen gas for the sheath and auxiliary gas were set 65 and 8 arbitrary units respectively. The spray voltage was set to 4.5kV and the heated capillary temperature maintained at 250°C. The precision level of the instrument was ±0.02%.

**Supplementary Figure 1: Dose-dependent effects of various concentration of hot water extract of (A & B) *H. fomes* bark, (F) P-1 fraction, (G) P-2 fraction and (H) Quercitrin on LDH release from BRIN-BD11 cells, (C) Acarbose on starch digestion, (D) Sitagliptin on DPP-IV enzyme activity and (E) Guar gum on glucose diffusion *in vitro***

Values are Mean±SEM with n= 4. *P<0.05, **P<0.01 and ***P<0.001 compared to control alone.
